# Osmolality of donor human milk rises dramatically within minutes of fortification with varied degrees depending on the fortifier used

**DOI:** 10.3389/fped.2025.1596255

**Published:** 2025-06-06

**Authors:** Qin Tang, Dingding Yin, Zhenchao Jin, Lian Zhang, Ping Zhou

**Affiliations:** ^1^ Department of Neonatology, Baoan Women’s and Children’s Hospital, Shenzhen, China; ^2^ Donor Milk Bank, Baoan Women’s and Children’s Hospital, Shenzhen, China

**Keywords:** donor human milk, human milk fortifier, osmolality, protein fortifier, preterm infants

## Abstract

**Background:**

The addition of fortifiers to human milk can enhance its energy and protein content, although it results in an increase in osmolality, which may vary depending on the composition of fortifiers. The manner in which osmolality changes over time remains uncertain.

**Aims:**

We hypothesized that the impact of different fortifiers on osmolality would vary significantly and evolve dynamically with the duration of place time.

**Methods:**

Osmolality measurements were taken from the donor human milk (DHM)at 3 and 22 h after the addition of six different human milk fortifiers (HMFs) using a freezing osmolality meter. Furthermore, the osmolality was evaluated at nine time points following the standard fortification procedure with HMF1-3.

**Results:**

(1) The mean osmolality of the unfortified donor milk was 299 mOsm/kg. The addition of three multi-component fortifiers (HMF1-3) led to a significant elevation in osmolality (*P* < 0.05), albeit to varying extents (54.6–109.1 mOsm/kg). The addition of the preterm formula HMF4 resulted in a lesser increase in osmolality in comparison to HMF1 and HMF2. (2) The osmolality increased significantly by 183.0 ± 27.4 mOsm/kg after the addition of the protein fortifier PF1, whereas it increased by only 8.9 ± 2.9 mOsm/kg after the addition of PF2 (*P* < 0.05). (3) The osmolality at 22 h showed a minimal increase of 0.3–3.7 mOsm/kg (0.1%–1.0%) compared to the osmolality at 3 h following the addition of the six fortifiers. (4) The increase in osmolality following fortification with HMF1-3 was predominantly observed within two minutes of addition, accounting for 85.9%–91.2% of the total increase, followed by a slow increase over the subsequent 12 h, with a slight decrease thereafter.

**Conclusions:**

The addition of fortifiers significantly increased the osmolality of DHM. However, the degree of increase varied depending on the nutrient composition and content of the fortifiers used. It remains a challenge to avoid the rapid increase in osmolality of DHM within a very short time after the addition of a fortifier, even when fortifying at the bedside.

## 1 Introduction

Optimal nutrition is vital for fulfilling the growth and developmental requirements of preterm infants, exerting a pivotal influence on their short- and long-term prognosis (1). In order to prevent extrauterine growth retardation and to improve clinical outcomes, it is standard practice to supplement with a human milk fortifier (HMF) in human milk when managing the enteral feeding of very preterm infants (2). There are numerous sources and forms of HMF, with the most prevalent being multi-component powder or liquid fortifiers derived from cow's milk, as well as fortifiers derived from donor human milk and single-component fortifiers (3). The availability of these fortifiers varies across countries. To illustrate, in the United States, liquid multi-component fortifiers and fortifiers derived from donor milk are the predominant forms (4), whereas only powdered multi-component fortifiers are available in China. In some resource-limited countries, the use of a preterm formula as a fortifier has been demonstrated to be equally effective as a multi-component HMF (5).

Fortifying human milk increases its osmolality due to the addition of protein and other nutrients (6). This elevated osmolality may have adverse effects on preterm infants, including delayed gastric emptying, disruption to intestinal mucosal integrity, increased incidence of gastroesophageal reflux, feeding intolerance, and necrotizing enterocolitis (7–9). In order to circumvent these potential complications, the American Academy of Pediatrics (AAP) has advised that the osmolality of enteral nutrition should not exceed 450 mOsm/kg (10). Several studies have documented considerable variability in the elevation of osmolality when diverse HMFs are introduced, with some exceeding the AAP-recommended thresholds (11–14). Consequently, osmolality may be regarded as a predictable factor in the clinical selection of HMF. However, there is a paucity of systematic comparative studies examining the impact of addition of diverse fortifiers, including protein fortifiers or infant formula as fortifiers, on the osmolality of human milk.

Furthermore, the timing of the supplementation with HFM is also inconsistent. Some studies have indicated that the osmolality of human milk increases over time following the addition of a fortifier (13, 14). It has therefore been recommended that the fortifier be used immediately after its addition at the bedside. However, other studies have not supported this approach (15, 16). Consequently, this *in vitro* study was designed to compare the difference in osmolality of donor human milk (DHM) with the addition of different fortifiers and to observe the trend in osmolality of fortified human milk with prolonged placement time.

## 2 Methods and materials

### 2.1 Study subjects

#### 2.1.1 Donor and donor human milk

Between March 2022 and December 2022, thirty healthy mothers donated milk and 70 ml of milk was provided by each donor, which was subsequently divided into seven portions of 10 ml each for testing. The study was approved by the Ethics Committee of Baoan Women's and Children's Hospital (LLSC-2021-04-01-01-KS), and all donors provided written informed consent prior to participation.

#### 2.1.2 Fortifiers

The study employed six human milk fortifiers, comprising three commercially available multi-component fortifiers (HMF1, HMF2, and HMF3), one preterm infant formula utilized as a fortifier in resource-limited countries (HMF4) (5), one liquid protein fortifier (PF1), and one powdered protein fortifier (PF2). All six fortifiers are cow-milk-derived. The macronutrient and micronutrient compositions of the six fortifiers are presented in Table 1. The data were derived from the ingredient labeling of the products and converted uniformly to content per gram (PF1 in liquid form is expressed as content per ml).

**Table 1:** Main nutrient composition of Cow milk-derived fortifiers (per gram).

Nutrients	Multi-component fortifier		Preterm formula		Protein fortifier	
	HMF1	HMF2	HMF3	HMF4	PF1^a^	PF2
Energy (kcal)	4.35	3.89	4.92	4.98	0.68	3.57
Protein (g)	0.36	0.28	0.39	0.14	0.17	0.86
Type hydrolyzed	Whey partial	Whey intact	Casein/Whey partial	Whey partial	Casein deep	Whey intact
Fat (g)	0.18	0.10	0.35	0.25	–	–
Carbohydrates (g)	0.32	0.50	<0.14	0.51	–	–
Sodium (mg)	9.15	4.44	5.63	2.69	–	2.14
Potassium (mg)	12.10	17.8	9.15	6.05	–	–
Calcium (mg)	18.90	32.20	28.50	7.60	–	–
Iron (mg)	0.45	0.09	0.46	0.09	–	–
Vitamin A (IU)	277	172	352	40	–	–
Vitamin D (IU)	35	33	59	5	–	–

HMF1, PreNAN FM85® (Nestle); HMF2, Similac® (Abbott); HMF3, Enfamil® (Mead Johnson); HMF4, PreNAN PDF (Nestle Deutschland AG); PF1, Liquid Protein Fortifier (Abbott); PF2, Beneprotein® (Nestle).

^a^ PF1 presented as 1 ml containing 0.17 g of protein.

### 2.2 Research methods

#### 2.2.1 Collection, storage, and composition analysis of donor human milk

Each donor provided 70 ml of DHM, which was collected and stored in polyethylene bottles. The milk was pasteurized and then stored in a refrigerator set at −20°C for 30 days. Prior to analysis, the donor milk was thawed at room temperature and homogenized using ultrasonic vibration (1.5 s/ml of human milk, MIRIS Ultrasonic Processor, Sweden). The macronutrient composition was analyzed respectively using infrared transmission spectrometry (MIRIS HMA, Sweden).

#### 2.2.2 Preparation of fortified donor human milk

The thawed donor milk from each donor was divided into seven 10 ml portions. One portion was designated as the control, while three portions were fortified with three multi-component fortifiers (HMF1-3) in accordance with the product instructions for 100 ml of DHM, with fortification amounts of 4 g, 3.6 g, and 2.84 g, respectively. A further portion was fortified with 4 g of HMF4 for 100 ml of DHM, as previously described in the literature (5). Two further portions were subjected to compositional analysis in order to determine the protein content and were then fortified with two different protein fortifiers (PF1 and PF2) in order to achieve the optimal protein content of human milk for preterm infants. This was determined to be 2.7–3.3 g/100 ml, in accordance with the recommendations of the European Society of Paediatric Gastroenterology, Hepatology and Nutrition (17). The powdered fortifier was weighed with a precision electronic scale with an accuracy of 0.01 g, and the liquid fortifier was accurately aspirated with a medical syringe. After adding the fortifier, DHM was manually mixed for one minute.

#### 2.2.3 Measurement of osmolality

The osmolality was measured by an osmometer (YASN Osmo310, UK) utilizing the freezing-point method. Prior to measurement, calibration was carried out using a standard solution. Each sample was measured twice at each time point, and the results were averaged. The osmolality of unfortified DHM and fortified DHM with six different fortifiers was measured at 3 and 22 h, which is consistent with the typical consumption of first and last milk in preterm infants. The samples were stored in a refrigerator at 4°C between the two measurements.

To investigate the changes in osmolality of fortified breast milk over placement time, the osmolality of DHM fortified with HMF1, HMF2, and HMF3, respectively, was measured at nine time points (2 min, 5 min, 10 min, 30 min, 1 h, 3 h, 6 h, 12 h, and 22 h), and the trends were plotted.

### 2.3 Statistics

Statistical analyses were performed using the R programming language. The normality of osmolality and nutrient measurements was evaluated at all time points. In the case of non-normally distributed measures, the median and interquartile range were used as means of description, whereas normally distributed measures were described using the mean ± standard deviation. The Mann–Whitney non-parametric test was employed to ascertain any significant differences between non-normally distributed parameters, whereas the *t*-Student test was utilized for normally distributed parameters. A statistically significant difference was deemed to have occurred when the *p*-value was less than 0.05.

## 3 Results

### 3.1 Basic characteristics

The mean age of the donors was 30 years, with 73.3% of them being first-time deliveries. The median gestational age was 35.5 weeks (26^+4^–41^+3^ w), with the median number of days that donor milk was obtained being 41 days. The content of macronutrients in unfortified DHM was presented in Table 2, and there was no significant difference in macronutrients DHM at 3 and 22 h.

**Table 2:** General information of donors and composition of donor milk.

Basic information of donor	Median [IQR], *n* = 30		
Age, years	30.00 [28.00, 31.00]		
Lactation time, days	41.00 [30.75, 64.50]		
Primipara (*n*, %)	22/30 (73.3%)		
Gestational age, (*n*, %)			
<28 weeks	2/30 (7%)		
28–36^+6^ weeks	13/30 (43%)		
≥37 weeks	15/30 (50%)		
Macronutrients in DHM	3 h	22 h	*P*
Energy, kcal/dl	71.0 ± 7.1	70.6 ± 7.1	0.79
Protein, g/dl	1.3 ± 0.3	1.3 ± 0.3	0.96
Fat, g/dl	3.4 ± 0.6	3.4 ± 0.7	0.88
Carbohydrates, g/dl	8.3 ± 0.3	8.2 ± 0.3	0.36

DHM, donor human milk; IQR, interquartile range.

### 3.2 Osmolality was significantly elevated by the addition of various fortifiers, although the extent of this increase varied considerably

**Figure 1:**
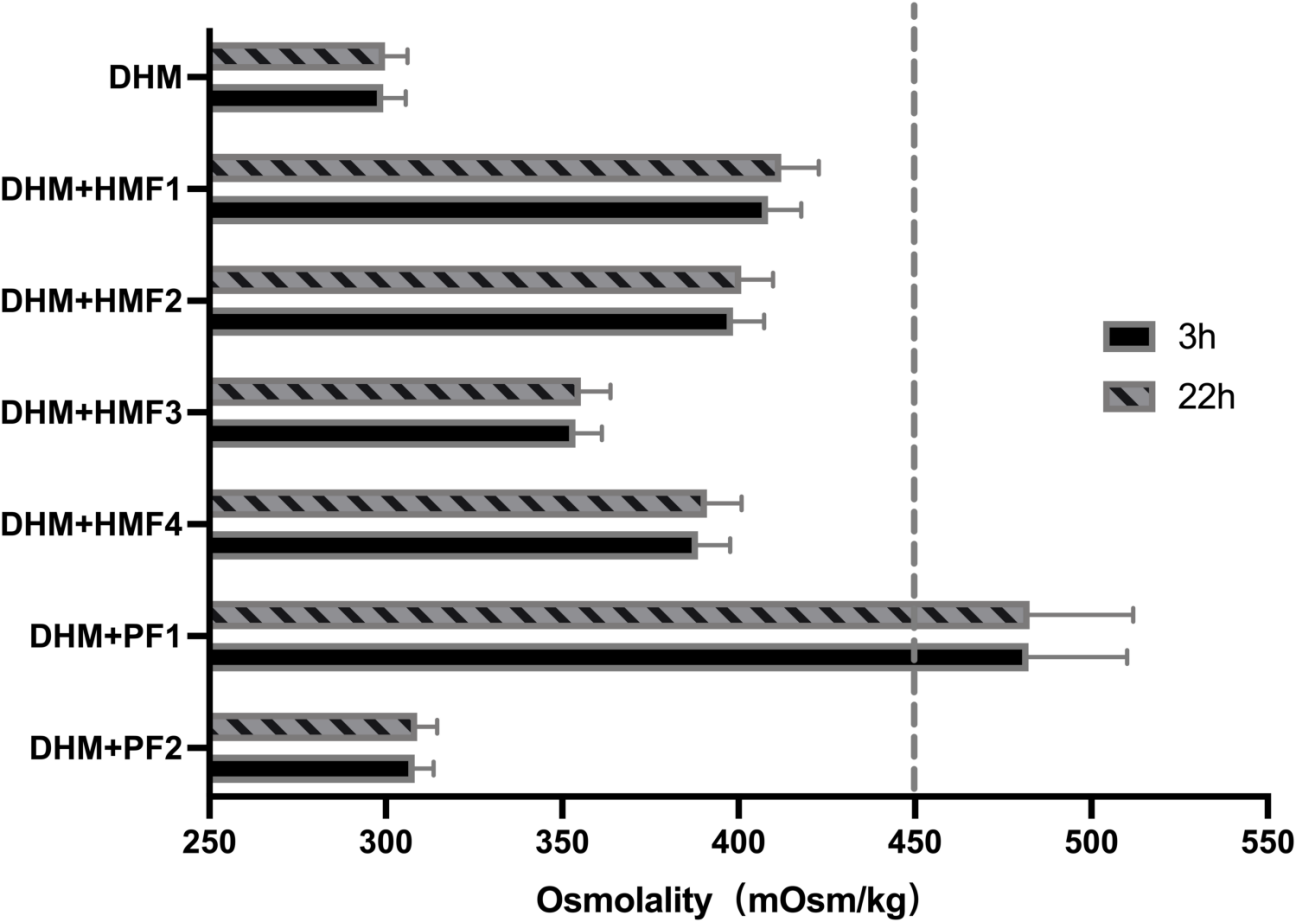
Osmolality of DHM fortified with different fortifiers. Compared with the unfortified DHM, the osmolality of DHM fortified with six different fortifiers was significantly increased (*P* < 0.001). There is no difference between 3 h and 22 h in the osmolality of unfortified DHM or DHM fortified with different fortifiers. DHM, donor human milk; HMF1-3, common used multi-component fortifiers; HMF4, preterm formula; PF1, liquid protein fortifier; PF2, powdered protein fortifier.

**Figure 2:**
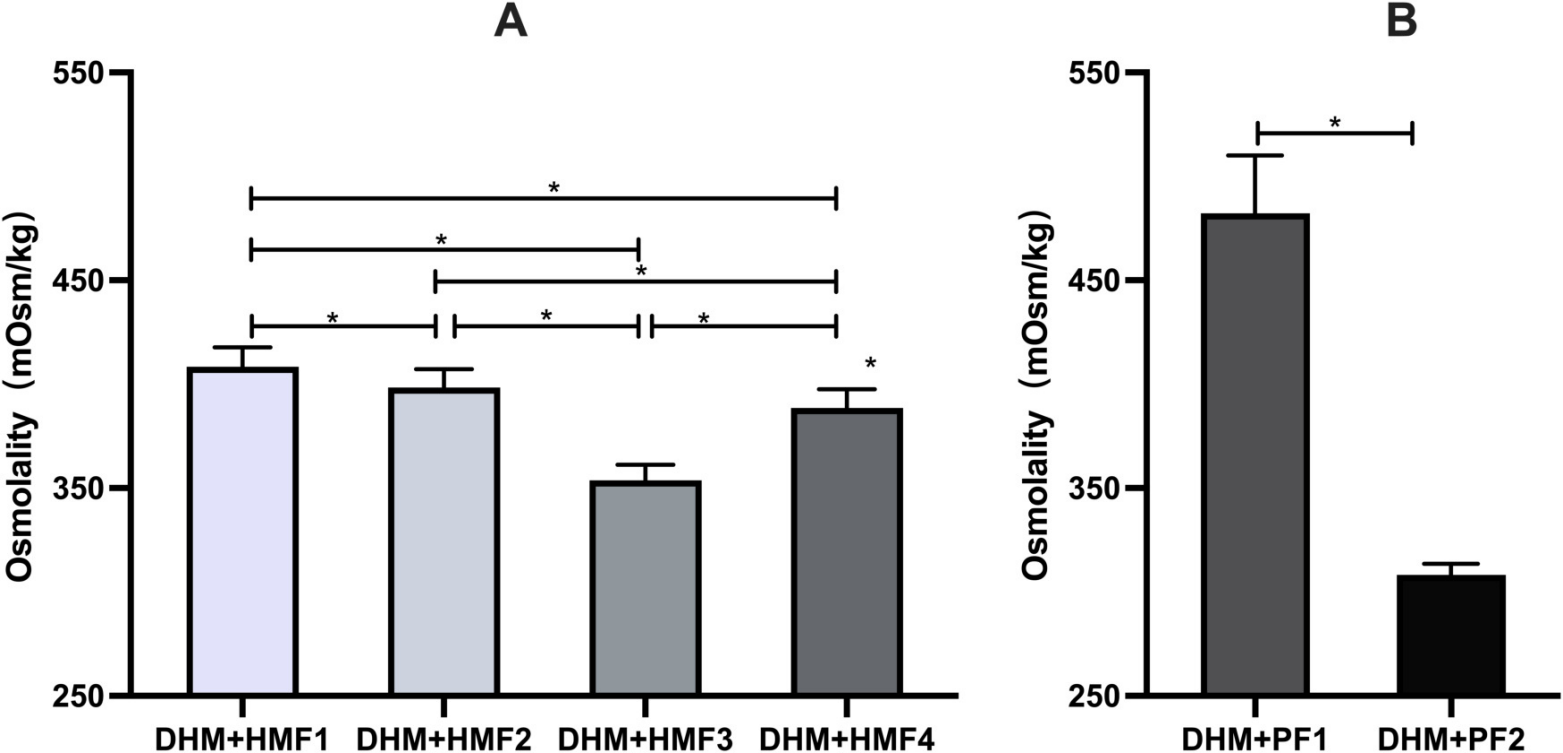
Comparison of osmolality on DHM fortified with multi-component fortifiers or protein fortifiers. **(A)** Significant differences in osmolality of DHM fortified with four multi-component fortifiers were found in the between-group comparisons, respectively; **(B)** Significant differences in osmolality of DHM fortified with two protein fortifiers. * indicates *p* < 0.01 between groups.

In comparison to unfortified DHM with an osmolality of 299 mOsm/kg (3 h) and 301 mOsm/kg (22 h), the addition of three commercially available multi-component fortifiers (HMF1-3) resulted in a significant increase in osmolality. The utilization of disparate fortifiers gave rise to considerable discrepancies in the extent of osmolality augmentation. A statistically significant difference (*p* < 0.05) was observed in the comparison of osmolality between HMF1, HMF2, and HMF3. However, none of the levels exceeded the recommended levels set forth by the AAP (Figure 1 and Table 3). Furthermore, the addition of preterm infant formula (HMF4) resulted in a statistically significant increase in osmolality too. A significant difference (*p* < 0.05) was observed between HMF4 and HMF1-3, with HMF4 exhibiting a higher osmolality than HMF3 and a lower osmolality than HMF1 and HMF2 (Figure 2A and Table 3).

**Table 3:** Comparison of osmolality and increased osmolality on DHM fortified with different fortifiers (mOsm/kg, *n* = 30).

Fortification group	Osmolality		Value added mOsm/kg (%)	P* (3 h vs. 22 h)	Increased osmolality		*P* (3 h vs. 22 h)
	3 h	22 h			3 h	22 h	
DHM^※^	299 (297, 305)	301 (299, 305.75)	1.5 (0.49)	0.211	–	–	–
DHM + HMF1	408.4 ± 9.4	412.1 ± 10.7	3.7 (1.00)	0.160	109.1 ± 6.1	111.4 ± 7.5	0.001^§^
DHM + HMF2	398.5 ± 8.8^†^	400.8 ± 9.0^†^	2.3 (0.58)	0.312	99.2 ± 4.9	100.1 ± 5.7	0.078
DHM + HMF3	353.8 ± 7.5^†^^,^^¶^	355.3 ± 8.4^†^^,^^¶^	1.5 (0.42)	0.467	54.6 ± 3.6	54.6 ± 4.5	0.947
DHM + HMF4	388.5 ± 9.1^†^^,^^¶^^,^^☨^	391.0 ± 9.8^†^^,^^¶^^,^^☨^	2.5 (0.64)	0.312	89.3 ± 5.1	90.3 ± 6.1	0.049^§^
DHM + PF1	482.2 ± 27.9	482.5 ± 29.3	0.3 (0.10)	0.967	183.0 ± 27.4	181.8 ± 28.7	0.063
DHM + PF2	308.2 ± 5.4^♯^	308.9 ± 5.7^♯^	0.8 (0.24)	0.594	8.9 ± 2.9	8.2 ± 3.3	0.096

^※^ Compared with the unfortified DHM, the osmolality of DHM fortified with six different fortifiers was significantly increased (*P* < 0.001).

* No difference between 3 h and 22 h in the osmolality of unfortified DHM or DHM fortified with different fortifiers.

^§^ After adding various fortifiers, the increased osmolality of DHM + HMF1 and DHM + HMF4 was significantly different between 3 h and 22 h (*P* < 0.05).

^†^ Compared to DHM + HMF1, *P* < 0.05.

^¶^ Compared to DHM + HMF2, *P* < 0.05.

^☨^ Compared to DHM + HMF3, *P* < 0.05.

^♯^ Compared to DHM + PF1, *P* < 0.05.

The addition of protein fortifier PF1 resulted in a statistically significant increase in osmolality, exceeding the APP-recommended value. In contrast, the osmolality of human milk exhibited a minimal increase of 8.93 ± 2.94 mOsm/kg when fortified with PF2 (*P* < 0.05) (Figure 2B and Table 3).

A comparison of the osmolality of unfortified DHM and DHM fortified with six different fortifiers at 3 h revealed that the osmolality at 22 h increased by a mere 0.3–3.7 mOsm/kg (0.10%–1.0%, *P* > 0.05). However, a statistically significant difference was observed between the increased values of osmolality at 3 and 22 h after fortification for HMF1 and HMF4 (Table 3).

### 3.3 The marked elevation in osmolality of DHM occurred in a very short time after fortifier addition

**Figure 3:**
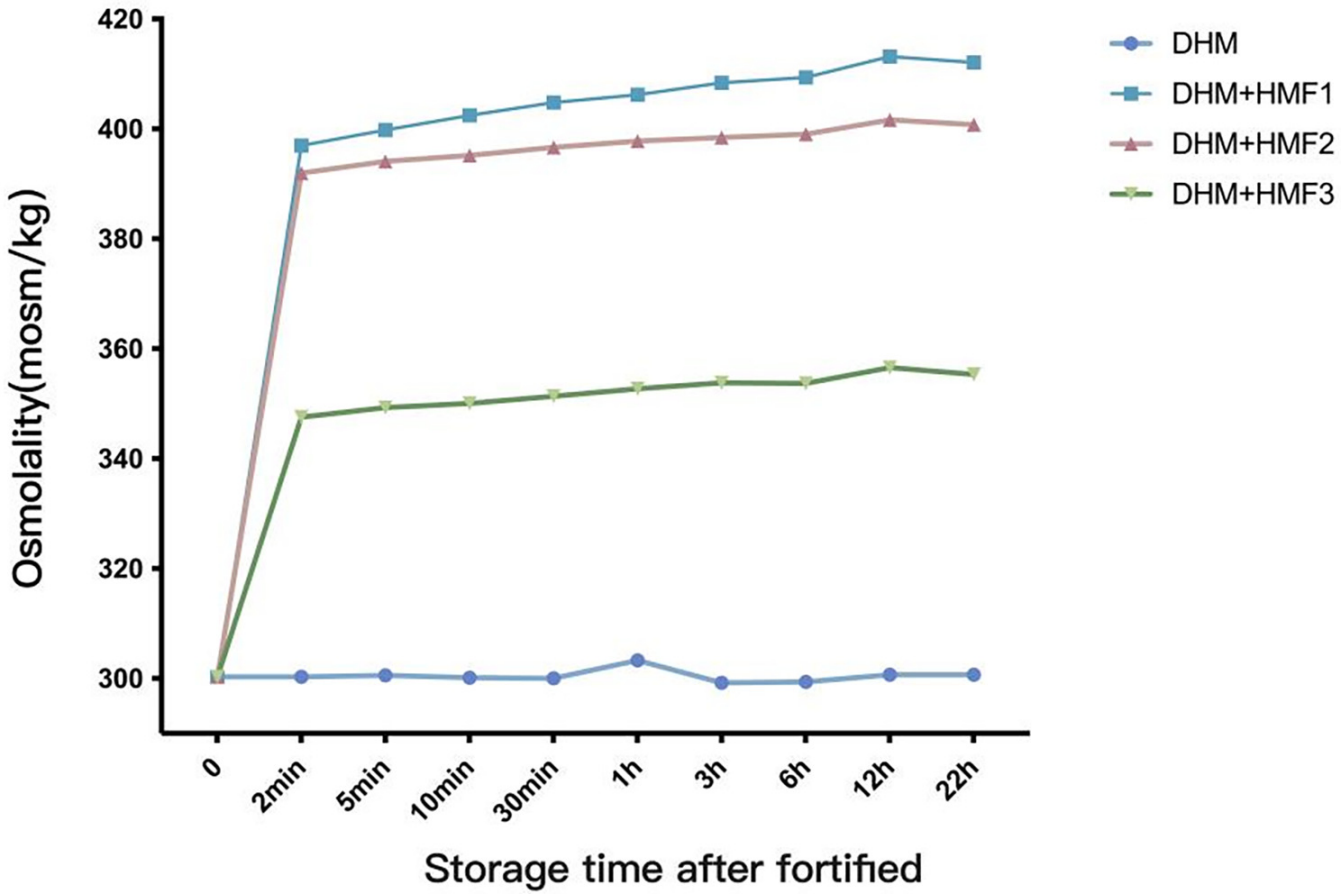
Trends in osmolality of DHM fortified with HMF1-3 over the placement time. DHM, donor human milk; HMF, human milk fortifier.

The osmolality of fortified DHM increased rapidly after the addition of three different fortifiers (HMF1-3). The increase in osmolality was observed to occur between 85.9% and 91.2% within two minutes of addition. This was followed by a slow increase over the subsequent 12 h, which then declined slightly from 12 to 22 h (Figure 3 and Table 4).

**Table 4:** The altered value and proportion of increased osmolality at seven intervals of nine time points following the fortification of DHM with HMF1-3 (mOsm/kg, %).

Fortification group	Increased osmolality: value (mOsm/kg) and proportion (%)						
	0–2 min	2–5 min	5–10 min	10 min–1 h	1–3 h	3–12 h	12–22 h
DHM + HMF1	96.7 (86.50)	2.8 (2.50)	2.7 (2.41)	3.8 (3.40)	2.1 (1.88)	4.8 (4.29)	−1.1 (−0.98)
DHM + HMF2	91.7 (91.20)	2.1 (2.10)	1.1 (1.09)	2.6 (2.60)	0.7 (0.70)	3.16 (3.13)	−0.8 (−0.82)
DHM + HMF3	47.2 (85.97)	1.8 (3.27)	0.7 (1.30)	2.6 (4.74)	1.1 (2.00)	2.8 (5.10)	−1.3 (−2.36)

DHM, donor human milk; HMF1-3, common used multi-component fortifiers; min, minute; h, hour.

## 4 Discussion

The study demonstrated that the addition of a fortifier to DHM resulted in a significant elevation in the osmolality of the milk. The extent of this increase varies depending on the specific brand and type of fortifier employed. The significant elevation in osmolality occurred in a very short period after fortifier addition.

At present, a range of fortifiers are available globally, with different usage habits observed across countries, reflecting varying levels of accessibility to fortifiers. The incorporation of additional protein fortifiers has become a fundamental aspect of the individualized fortification method (18). Therefore, the alteration in osmolality resulting from the utilization of disparate fortifiers represents a significant concern. The vehicle for this study was donor milk, which has an osmolality similar to that of fresh human milk (19). The mean osmolality of human milk exhibited a statistically significant increase when three commonly utilized multi-component fortifiers were supplemented. However, none of the fortifiers exceeded the recommended values set out by the APP and this is an element supporting the safety of fortification of human milk. The lowest observed increase in osmolality was associated with the addition of HMF3, which may be attributed to its relatively low carbohydrate content. Amylase in human milk can catalyze the enzymatic breakdown of added carbohydrates into monosaccharides or oligosaccharides, thereby increasing the absolute solute load and resulting in an expected osmolality increase of 20 mOsm/kg with the addition of 1 g of carbohydrate to 100 ml of human milk (20). The use of preterm infant formula as a HMF resulted in a comparatively minor increase in osmolality in comparison to the majority of dedicated multi-component fortifiers. This may be due to the lower protein content in the formula (21). Consequently, the effect of fortification on osmolality is closely linked to the composition and content of the nutrients involved. An interesting alternative could be donkey's milk as a basis for fortification, but the availability of donkey's milk is limited, making this option less viable on a large scale, and this is why we didn't analyze donkey's milk (22).

The ESPGHAN suggests that an ideal protein content of 2.7–3.3 g/100 ml of human milk would be sufficient to meet the rapid growth needs of preterm infants (17). However, the majority of human milk with standard fortification fails to meet this requirement. Accordingly, individualized fortification has emerged as a potential solution to enhance the protein-energy intake ratio by supplementing with additional protein (4, 23–25). However, Kreissl et al. (6) found that the osmolality could reach as high as 600 mOsm/kg with the addition of more protein, indicating the necessity for caution. The efficacy on osmolality of two protein fortifiers, PF1 (liquid, containing only deeply hydrolyzed casein) and PF2 (powdered, containing non-hydrolyzed whey protein), was evaluated, and a significant difference in increased osmolality was observed in this study. This discrepancy may be due to the differing protein types and degrees of hydrolysis. Choi et al. (20) found that the addition of 1 g of whey protein and 1 g of hydrolyzed protein to 100 ml of breast milk resulted in an average increase in osmolality of 4 and 38 mOsm/kg, respectively. The increase in osmolality was found to be linearly related to the dose, indicating that the degree of protein hydrolysis is also a critical factor in the process. It seems reasonable to posit that the higher the proportion of peptides or amino acids in protein hydrolysis, the greater the increase in solutes and, consequently, osmolality. While the AAP recommendations are based on empirical consensus and the latest guidelines did not specify an upper osmolality limit for enteral feeding (17), it is important to recognize that high osmolality remains a risk to the neonatal gut. It may, therefore, be advisable to utilize non-hydrolyzed protein-based HMF in very preterm infants with immature intestines and to select a fortifier with a lower osmolality.

The osmolality increase observed in this study, using DHM as the study vehicle, was found to be 30–50 mOsm/kg lower than that previously measured using fresh breast milk (13). Moreover, the osmolality increase following the prolonged storage of fortified donor milk up to 22 h was found to be only 0.3–3.7 mOsm/kg, a markedly smaller value than the 10–40 mOsm/L observed in previous studies (6, 7, 20). This may be attributed to the heat treatment during pasteurization of donor milk, which denatures the protein and reduces the amount of solute present in breast milk. Furthermore, the partial inactivation of catalase, lipase, and amylase in DHM after pasteurization resulted in a diminished degradation of macronutrients in the fortification, which in turn led to a smaller increase in osmolality (26). Therefore, it should be noted that if the fortifier is added to breast milk, the increased osmolality may be higher than that measured in DHM. Additionally, caution is needed, as the measured osmolality may exceed the values indicated on the label. The addition of HMF1 to DHM resulted in an osmolality of 408.37 mOsm/kg, which is higher than the value stated on the label (381 mOsm/kg). Such a difference may be related to inherent variations in the composition of the batch or the quantity of the product dispensed.

In clinical settings, there are two methods for the administration of fortifiers: centralized addition and storage and bedside addition before feeding and immediate use. The former method entails the dispensing of fortifiers in a centralized manner, in accordance with hygienic protocols, subsequent to precise weighing. This approach helps to avoid wastage of breast milk and fortifiers while simultaneously reducing the risk of contamination. Nevertheless, some studies advocate for the immediate addition of a fortifier at the bedside, as the osmolality of human milk tends to increase over time due to the hydrolysis of carbohydrates (15, 27). The findings of our study revealed that between 85.88 and 91.21% of the osmolality increase occurred within two minutes following the addition of fortifier. This result is consistent with that reported by Nathalie (16), who found that over 70% of the osmolality increase occurred within one minute of fortification. Subsequently, there was a gradual increase in osmolality during the storage period. It is challenging to prevent the rapid increase in osmolality over a short period of time, even with immediate bedside additions. The subsequent minor increase in osmolality is clinically inconsequential (6, 28). It is therefore imperative to select a fortifier with an appropriate composition and content in order to avoid elevated osmolality following the fortification process. It is important to acknowledge the potential risks associated with the addition of fortifiers at the bedside, including inaccurate weighing, inadequate fortifier dissolution, and contamination. Furthermore, our findings indicate that the osmolality of fortified DHM decreases after 12 h of storage at 4°C, which is consistent with the findings of previous studies (29). This decrease may be attributed to the aggregation of casein molecules during low-temperature storage.

The study may facilitate the rational selection and utilization of fortifiers in clinical settings; however, it is important to consider the limitations of the study. Firstly, the study did not examine the specific correlation between the main components and contents of fortifiers and osmolality. Secondly, although the level of increased osmolality varies significantly depending on the fortifier used, it is unclear whether this difference in osmolality affects the clinical outcome of the gastrointestinal tract in preterm infants, such as feeding tolerance or NEC, which requires further investigation. Moreover, quantification and comparison of the osmolality following the addition of human milk-derived fortifiers were not performed due to a shortage of sources.

## 5 Conclusions

The addition of a fortifier has a marked effect on the osmolality of DHM. However, the extent of the increase in osmolality varies considerably depending on the nutrient composition and content of the fortifier in question. It can be challenging to prevent a rapid increase in osmolality within a very short period of time, even when fortifying at the bedside. Therefore, in clinical practice, it is of the utmost importance to consider the feeding tolerance of preterm infants and the degree of osmolality increase when selecting the most appropriate fortifier.

## Data Availability

The original contributions presented in the study are included in the article/Supplementary Material, further inquiries can be directed to the corresponding author.
